# Friedewald formula may be used to calculate non-HDL-C from LDL-C and TG

**DOI:** 10.3389/fmed.2023.1247126

**Published:** 2023-09-14

**Authors:** Jerzy Romaszko, Leszek Gromadziński, Adam Buciński

**Affiliations:** ^1^Department of Family Medicine and Infectious Diseases, School of Medicine, Collegium Medicum, University of Warmia and Mazury in Olsztyn, Olsztyn, Poland; ^2^Department of Cardiology and Internal Medicine, School of Medicine, Collegium Medicum, University of Warmia and Mazury, Olsztyn, Poland; ^3^Department of Biopharmacy, Faculty of Pharmacy, Ludwik Rydygier Collegium Medicum in Bydgoszcz, Nicolaus Copernicus University in Toruń, Bydgoszcz, Poland

**Keywords:** Friedewald formula, non-HDL-C, cost reduction, SCORE2, lipid panel

## Abstract

**Background:**

The Friedewald formula (FF) was originally designed 50 years ago and has been in use to this day despite better methods for estimating LDL cholesterol (LDL-C). Its success was mainly due to its simplicity. Nowadays most laboratories determine or can determine LDL-C by the direct method. The SCORE2 tables, recommended by the European Society of Cardiology, are based on non-HDL cholesterol (non-HDL-C). To calculate its value, one needs to know the values of total cholesterol (TC) and HDL-C. The presented idea is to use the FF to calculate non-HDL-C based on the values of LDL-C and TG instead of TC and HDL-C.

**Methods and findings:**

Based on database of 26,914 laboratory results, covering the complete lipid panel, the error regarding non-HDL-C values calculated in both ways (recommended and proposed) was determined. The average error in the LDL-C value calculated with the FF compared to the LDL-C value measured in the laboratory is 9.77%, while for non-HDL-C the error between the calculated and laboratory-determined value amounts to 8.88%. The proposed transformation of the FF also yields a much lower percentage of error calculations. Both LDL-C and non-HDL-C (calculated) in our material are strongly correlated with LDL-C and non-HDL-C (measured) values of *r* = 0.965 (*p* < 0.000) and *r* = 0.962 (*p* < 0.000), respectively.

**Conclusion:**

Non-HDL-C may be calculated based on the values of LDL-C and TG (without the need to determine the levels of TC and HDL-C). The proposed calculation may greatly reduce the cost of testing, given the price of a complete lipid profile.

## Background

The Friedewald formula (FF), due to its simplicity, has become an essential tool for estimating the level of LDL cholesterol (LDL-C) (1). Despite some objections regarding its accuracy and limitations of its use to situations when the level of triglycerides (TG) is below 400 mg/dL, for many local laboratories and for everyday needs in routine GP practice, the FF is sufficient and cost-free. Its accuracy is estimated to be about 80% (2).

The SCORE tables, recommended by the European Society of Cardiology, in their current version (SCORE2 and SCORE2-OP) are based on non-HDL cholesterol (non-HDL-C) (3).

We calculate non-HDL-C based on the values of total cholesterol (TC) and HDL-C.

(In the equations presented below, for the sake of the simplicity of presentation, abbreviations without “-C” are used, e.g. non-HDL-C is presented as NHDL, units in mg/dL):


NHDL=TC−HDL

$$\mathrm{LDL}=\mathrm{TC}-\mathrm{HDL}-\frac{1}{5}\mathrm{TG}$$

i.e.:


$$\mathrm{LDL}+\frac{1}{5}\mathrm{TG}=\mathrm{TC}-\mathrm{HDL}$$

and thus,


$$\mathrm{NHDL}=\mathrm{LDL}+\frac{1}{5}\mathrm{TG}$$

Although mathematically this is obvious (perhaps that is why no one has addressed it thus far), in a situation when only a section of the lipid panel (LDL-C and TG) is available, this calculation makes it possible to disregard other assessments (as long as TG < 400 mg/dL).

The aim of our research was the statistical verification of the above mathematical equation.

## Methods

Based on database of 26,914 laboratory results (anonymized, no patient data included), including the complete lipid profile, with LDL-C determined by laboratory tests, the error regarding non-HDL-C values calculated in both ways (recommended and proposed by us with the transformed formula) was determined. The error in the value of LDL-C calculated with the Friedewald formula and laboratory-determined LDL-C was also evaluated similarly. The data for analysis were collected from the laboratory of the “Diagnostyka” network and the Provincial Specialist Hospital in Olsztyn (Poland). These certified laboratories determine LDL-C (and other lipid parameters) using the colorimetric-enzymatic method (Roche/Hitachi cobas 6000). These laboratories perform the quality control of assays on a daily basis and the permissible measurement error for LDL-C is 10%. Data analyzed in the study were collected in the period from 01.01.2021 till 08.05.2023. Researchers gained access to the data for research purposes on 10.05.2023. Statistical analyses were performed with the employment of Statistica 13.3 (TIBCO Software Inc.). Abundance, mean value, standard deviation, median, and percentages were calculated for all study variables. The Pearson correlation coefficient (*r*^2^) was used to assess the association between variables.

## Results

The average error regarding the LDL-*C*_F_ value calculated with the FF as compared to the LDL-*C*_L_ value measured with the laboratory test is 9.77% in the analyzed study material (TG <400 mg/dL). Interestingly, the error in calculating non-HDL-*C*_F_ (with the transformed Friedewald formula) is smaller than the error in calculating LDL-*C*_F_, and amounts to 8.88%. The results are summarized in Table 1.

**Table 1:** Comparison of mean values of LDL-*C*_L_ and LDL-*C*_F_, mean values of non-HDL-*C*_L_ and non-HDL-*C*_F_, and percentage of erroneous measurements.

	LDL-C mg%			Non-HDL-C mg%		
	Laboratory avg. (SD; median)	Calculated avg. (SD; median)	Error (%) avg. (SD; median)	Laboratory avg. (SD; median)	Calculated avg. (SD; median)	Error (%) avg. (SD; median)
Total (*n* = 26,914)	108.61 (44.62; 104)	97.94 (43.18; 98.20)	−9.77 (31.63; 9.24)	124.39 (48.06; 118.00)	135.06 (52.33; 128.80)	8.88 (8.84; −8.18)
Underestimate <10%		43.84% (*n* = 11,800)			8.33% (*n* = 2,242)	
Error of the absolute value >25%		6.71% (*n* = 1,806)			3.92% (*n* = 1,055)	
Overestimate <10%		7.43% (*n* = 1,999)			50.11% (*n* = 13,487)	
TG <400 mg% (*n* = 26,516)	108.56 (44.52; 104.00)	98.29 (42.41; 93.20)	−9.07 (17.85; 9.17)	123.12 (45.82; 117.00)	133.39 (48.56; 127.80)	8.74 (8.53; −8.13)
Underestimate <10%		44.35 (*n* = 11,761)			8.35 (*n* = 2,214)	
Error of the absolute value >25%		5.94 (*n* = 1,574)			3.14 (*n* = 813)	
Overestimate <10%		7.48 (*n* = 1,983)			51.25 (*n* = 13,275)	

^*^Error—the difference expressed as a percentage of the laboratory value.

The proposed transformation of the Friedewald formula also yields a much lower percentage of erroneous calculations with an absolute value of more than 25% (5.94 vs. 3.14).

**Figure 1:**
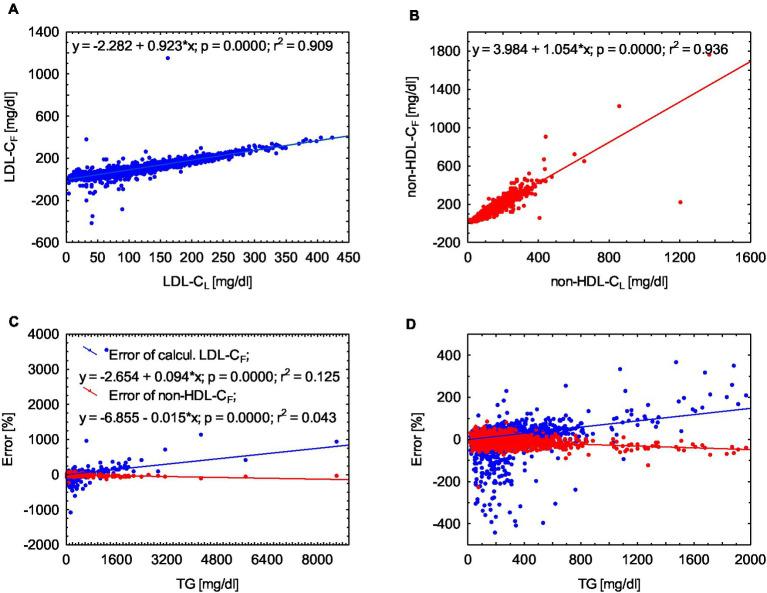
Pearson correlation of LDL-*C*_L_ and LDL-*C*_F_ (chart **A**) and for non-HDL-C (chart **B**). Error of estimated parameters expressed as a percentage of the laboratory value (chart **C**) and the enlarged fragment of the presented graph C (chart **D**), TG not limited.

Both LDL-*C*_F_ and non-HDL-*C*_F_ in our material are strongly correlated with LDL-*C*_L_ and non-HDL-*C*_L_ values of *r*^2^ = 0.909 and *r*^2^ = 0.936, respectively, (or *r*^2^ = 0.931 and *r*^2^ = 0.925 when TG <400 mg/dL) and the *p*-value is <0.0001 in each calculation (Figure 1).

The equation we propose allows for obtaining non-HDL-*C*_F_ values that are closer to laboratory values than those attainable with the use of the classic Friedewald formula for LDL-*C*_F_.

The comparison is even more interesting when we use the absolute value of the mean error, expressed as a percentage of the laboratory value, in determining LDL-*C*_F_ and non-HDL-*C*_F_. Here a statistically significant difference (*p*-value <0.0001) occurs for any value of TG and those limited below 400 mg/dL.

## Discussion

Before using our data for the suggested equation, we validated it by calculating LDL-*C*_F_ and we obtained the correlation with LDL-*L*_L_ values comparable to that of other authors (not worse) (Figures 1A,B) (4, 5). However, for our discussion, it is significant that the determination of non-HDL-*C*_F_ yields closer values (smaller error) than those resulting from calculating LDL-*C*_F_ (Figures 1C,D). Moreover, the Friedewald formula tends to underestimate LDL-*C*_F_ (the findings consistent with literature) while non-HDL-*C*_F_ is overestimated (Table 1) (6). This overestimation may result in unnecessary therapeutic interventions, while the underestimation of LDL-*C*_F_ will lead to the lack of therapy whatsoever with obvious consequences (7). Evaluating the consequences of both errors obviously requires further research, but at first glance it seems that an error in the determination of non-HDL-*C*_F_ should have far less significant consequences.

The Friedewald formula (FF) was originally designed 50 years ago and has been in use to this day despite better methods for estimating LDL-C (8, 9). Its success was driven by three components: ease of use, economy, and availability. In 2023, most laboratories determine or can determine LDL-C by the direct method. Still, the economic factor remains.

GPs, during their everyday work with chronically treated, previously comprehensively diagnosed patients, need LDL-C, TG and non-HDL-C values to make therapeutic decisions (10). TC and HDL values rarely influence such decisions, e.g., according to the ESC Guidelines on cardiovascular disease prevention in clinical practice of 2021, there are no specific goals for HDL-C (10, 11). Determination of LDL-C and TG and subsequent calculation of non-HDL-*C*_F_ reduce the cost of testing by more than 40% compared to the price of a complete lipid profile, or by almost 30% if LDL-C is calculated with the FF (estimated on the basis of test price lists of the 3 largest laboratory networks in Poland).

In 2021, GPs in Poland ordered 5,738,183 assessments of TC, 4,886,008 assessments of HDL-C and 5,143,444 assessments of TG (unpublished data obtained from the National Health Fund). Assuming that making the suggested formula widely recognizable and commonly used would contribute to the estimated reduction in the number of assessments of TC and HDL-C by 50%, and assuming that each test costs about 2 Euro we arrive at savings of roughly 10 million Euro annually. It should be remembered that 2021 (the only one for which we were able to obtain relevant data) was the year of COVID-19, and that our data do not include tests performed in the hospital setting and ambulatory specialist care. This underestimated extrapolation should be verified in a further study.

However, the practical application of the suggested method will probably initially be sporadic (incomplete lipid panel), but one may wonder whether it would not be reasonable, in routine medical practice, to consider the possibility of discarding multiple TC and HDL assessments.

Furthermore, an interesting argument would arise in a discussion with a patient with an anti-statin attitude, where the word cholesterol could be deliberately omitted by the doctor. However, this is rather a far-reaching digression requiring almost ideological changes, which are taking place but are not yet explicitly expressed.

## Conclusion

Non-HDL-C may be calculated based on the values of LDL-C and TG (without the need to determine the levels of TC and HDL-C). The equation: “non-HDL-C = LDL-C + 1/5 TG” allows for obtaining results closer to those of laboratory tests than the classic Friedewald formula for LDL. The proposed calculation may greatly reduce the cost of testing, given the price of a complete lipid profile.

### Limitations

The classic Friedewald formula may be employed irrespective of the patient’s age and sex. Latest algorithms based on, for example, neuronal networks use many more input data. However, in our opinion the great success of the original FF is related to its universality and simplicity rather than its accuracy (12). Hence in our study we only utilize completely anonymized laboratory results, with no indication of age and sex. Obviously, we plan to continue this research.

## Data availability statement

The original contributions presented in the study are included in the article/Supplementary material, further inquiries can be directed to the corresponding author.

## Ethics statement

The studies involving humans were approved by the Bioethics Committee of the Warmia and Mazury Regional Physicians’ Medical Chamber in Olsztyn, to which the authors are affiliated, on 09 May 2023 (L.Dz.WMlL-KB/147/2023). The studies were conducted in accordance with the local legislation and institutional requirements. Written informed consent for participation was not required from the participants or the participants’ legal guardians/next of kin in accordance with the national legislation and institutional requirements.

## Author contributions

JR was responsible for conceptualization, methodology, project administration, writing—original draft, and writing—review and editing. LG was responsible for data curation and writing—review and editing. AB was responsible for statistical analysis and writing—review and editing. All authors contributed to the article and approved the submitted version.

## Conflict of interest

The authors declare that the research was conducted in the absence of any commercial or financial relationships that could be construed as a potential conflict of interest.

## Publisher’s note

All claims expressed in this article are solely those of the authors and do not necessarily represent those of their affiliated organizations, or those of the publisher, the editors and the reviewers. Any product that may be evaluated in this article, or claim that may be made by its manufacturer, is not guaranteed or endorsed by the publisher.
